# Simulation of expansion and contraction for sudden plastic flow of Bingham cement grout and Newtonian fluids in a rectangular duct, using the lattice Boltzmann method

**DOI:** 10.1016/j.heliyon.2024.e28151

**Published:** 2024-03-16

**Authors:** José Luis Velázquez Ortega, Alberto Ignacio Guerrero Vergara

**Affiliations:** a Theoretical Research Center, National Autonomous University of Mexico – Faculty of Higher Studies Cuautitlan, Cuatitlan Izcalli, Mexico State, 54740, Mexico; b Engineering department, National Autonomous University of Mexico – Faculty of Higher Studies Cuautitlan, Cuatitlan Izcalli, Mexico State, 54750, Mexico

**Keywords:** Lattice Boltzmann, Cement grout, Bingham fluid, Newtonian fluids, Expansion and contraction

## Abstract

Bingham-type fluids are crucial in many industries and in geology. This study examines their behavior in reinforcing fractured rocks. Rheological properties were derived from experimental data of a water-cement mixture. Computational simulations were conducted using Lattice Boltzmann Method, with a modification to the relaxation parameter. Behavior of these mixtures in narrow ducts that widen and narrow suddenly, common in fractured rocks, was analyzed. Comparing Bingham and Newtonian fluids in various duct shapes provided insight into pressure distribution. Findings demonstrate that both cement-water mixtures, with or without addition of cement, adhere to Forchheimer flow patterns. Furthermore, it is observed that Newtonian fluids generate more intense vortices in expansive and contractive areas, resulting in higher pressure drops compared to Bingham plastics. The ultimate goal is to propose a predictive model based on mortar reinforcement for fractured rocks, taking into account rheological properties and water-cement ratio, thus reducing the need for costly experiments.

## 1 Introduction

Bingham fluids find practical applications in various industries, such as petroleum industries (e.g., drilling muds used for oil recovery), enhanced oil recovery, flow through fractal fracture networks, vortex chamber pumps for coal-water technologies, cement grouting in rock engineering, cluster magnetorheological finishing, toothpaste, dairy and food industries, biological fluids, emulsions, suspensions, and geophysical fluids [[1], [2], [3], [4], [5]].

An interesting application of Bingham fluids in the field of geotechnical engineering is related to the issue of rock fractures. Rock formations are damaged by fissures and joints that degrade their structural and mechanical properties. However, grout injection technologies can improve their strength and stability. They are also used in mines, dams, tunnels, slopes, and bridges. The degree of improvement depends on the mechanical properties of cement grout. The rheological properties of the grout, which are the key factors influencing injection design, determine its distribution in fractured rocks. These properties also affect the diffusion radius and reinforcement effect. Previous studies have shown that the rheological properties of the grout are influenced by its composition, water-cement ratio, and temperature. The ambient temperature at the injection site, which varies between 20 and 45 °C, affects the properties of the cement. In summary, the mechanical properties of cement grout influence the grout injection process [6].

Fiber-reinforced concrete (FRC) is extensively utilized in military, marine, and structural applications due to its durability and corrosion resistance. Jiang et al. [7] have shown that adding fiber to concrete enhances tensile, flexural, impact, fatigue, and abrasion strength, deformation capability, toughness, and load-bearing capacity after cracking. Grouting, a popular geotechnical process, seals underground water inflow and reinforces soil and rock masses in underground mining and development of underground spaces. Sui et al. [8] studied that sealing efficiency depends on factors such as fracture width, water speed, gel time, and grout volume. Initial water speed and aperture width are most influential.

Hydraulic fracturing, a popular oil and gas well stimulation method, creates high-permeability fractures in rock, enhancing fluid flow from reservoirs to wells. Success depends on accurately predicting fracture growth via numerical modeling during treatment design. Labrov [9] improved hydraulic fracturing models by modifying the power-law rheology to approximate Carreau rheology, providing a cost-effective closed-form solution for flow rate between parallel walls. This enhancement enhances accuracy in hydraulic fracturing simulations, particularly in small and large aperture areas. Grouting enhances rock mass tightness and reduces groundwater flow in rock engineering. Modeling grouting in fractures is crucial for effective design, especially due to strict demands for controlling groundwater flow in underground structures. Cement grouts are commonly treated as time-dependent Bingham fluids and injected through boreholes under constant pressure. Zoua et al. [10] developed a mathematical model for cement grout flow in rock fractures, considering two-phase flow with grout as a Bingham fluid and groundwater as a Newtonian fluid. Their study highlights the importance of the water phase in grouting, affecting pressure distribution and penetration.

Anchor bolt support represents a secure and efficient method of support within underground engineering applications. Wanga et al. [11] developed theoretical equations to calculate anchorage body interface shear strength before and after grouting and conducted field bolt-grouting tests on surrounding rock, analyzing grout diffusion under varying bolt lengths and effects of bolt-grouting parameters. Their findings inform bolt-grouting support design in fractured rock underground engineering.

The effective control of surrounding rock in underground engineering and mines is crucial for safety. Various methods such as bolt net support and grouting reinforcement are utilized, with grouting being particularly extensive due to deeper coal mining, preventing hazards like water inrush and roof collapse. Zhang et al. [12] introduced the theory of self-stress grouting reinforcement, developing the self-stress grouting material to enhance fractured rock mass reinforcement. Testing assessed swelling stress and expansive agent content, and an optimal cement-based grouting material was applied, improving the strength of surrounding rocks in mine roadways.

Low-permeability and heavy oil reservoirs are vital in petroleum recovery, with hydraulic fracturing significantly boosting oil well productivity and potentially leading to the development of fracture networks in multiple fractured wells, thereby enhancing production efficiency. Characterizing tight/shale reservoirs is challenging due to their complex fractures, which exhibit fractal patterns. Jianting Zhu's study [13] examines the flow of Bingham fluid in fractured reservoirs, taking into account both fluid properties and fractal characteristics. Fractures typically follow a power-law fractal distribution, with Bingham fluid flowrate increasing rapidly with pressure gradient, influenced by fracture size and distribution. Fractal parameters have varying effects on flow for Bingham and Newtonian fluids. Understanding water flow in fractured rocks is crucial for oil, gas extraction, and groundwater management, benefiting petroleum engineering. Sun et al. [14] introduced a truncated fractional-derivative model and used the SRT-LBM for seepage simulations to explore flow characteristics, revealing complexities in shear-thickening fluids. They also investigated shear stress memorization and its influence on flow equations, including the fractional-derivative critical Reynolds number.

The phenomenon of expansion and contraction refers to the flow separation that commonly occurs in pipelines when cross-sectional areas suddenly or gradually differ from one section to another. This results in a pressure drop, and depending on the flow velocity, this pressure drop can lead to vibrations and noise in the ducts. Flow separation and circulation play significant roles in numerous practical applications in food and chemical industries. This phenomenon is frequently encountered naturally in various industrial devices and equipment, including diffusers, turbine valves and blades, piping systems for cooling and air conditioning, flow devices used in the food and chemical industries, polymer manufacturing processes, pipes equipped with deflectors, high-performance heat exchangers, combustion chambers, and cooling passage in turbines [[15], [16], [17], [18]].

In 2009, Morales et al. [19] conducted a numerical simulation of three-dimensional laminar flow in a rectangular duct, for the sudden contraction of a Newtonian fluid. They analyzed different Reynolds numbers and aspect ratios. The results showed that the length and height of the region before the step, prior to separation, vary based on the Reynolds number and the aspect ratio. Furthermore, it is observed that the height of this region extends up to 76% of the total height of the step. An axial velocity profile corresponding to a fully developed flow was observed at the outlet. However, the velocity profile in the transverse direction did not develop fully even with an outlet length 60 times the height of the contraction.

Manica and De Bartoli [20] conducted a numerical study of an incompressible laminar flow in a channel with a 3:1 sudden expansion using power-law fluids. They employed an explicit three-stage Runge–Kutta finite difference scheme. Bifurcations, ranging from 0 to 2, were found within the power-law index values. In contrast, Moreno and Cervera [21] numerically modeled viscoplastic Bingham and Herschel–Bulkley fluids using the stabilized mixed velocity/pressure finite element method, by incorporating the regularized Papanastasiou viscoplastic model.

Another numerical study was conducted on the steady flow of Newtonian fluids during a two-dimensional 1:3 sudden expansion. The finite-volume method was used to solve the momentum and continuity equations by incorporating the power-law model within the laminar flow regime. The Reynolds number and power law index values were systematically varied to investigate the effects of shear thinning and thickening under slow-flow conditions. The findings revealed the existence of a redevelopment length, an additional pressure drop, and recirculation zones influenced by non-Newtonian viscous behaviors [22].

Wang and Ho [23] proposed the D2Q9 model for Bingham plastic fluids using the lattice Boltzmann method (LBM). Their proposal incorporated the effect of local shear rate into an equilibrium distribution function. For this simulation, they suggested a 2:1 expansion in a flat cavity, considering Bingham and Reynolds number ranges of 0 ≤ Bn ≤ 2000 and 0.2 ≤ Re ≤ 200, respectively. The results indicated that the model accurately predicted the deformation and corner vortex regions. Furthermore, the numerical values were consistent with those reported in the literature.

Ginzburg and Steiner [24] modeled the filling of viscoplastic and plastic metal alloys into expanding cavities using the LBM in two and three dimensions, respectively. They achieved this by combining Bingham's regularized model for viscoplastic fluids with an interface-free algorithm. These results were consistent with those obtained from the theoretical and numerical analyses.

Fusi et al. [25] and their collaborators investigated one-dimensional Bingham flows using pressure-dependent rheological parameters. They modified classical velocity profiles by assuming linear dependence of both viscosity and yield stress on pressure.

Tang et al. [26] utilized the LBM to simulate a Bingham fluid by incorporating a modified Papanastasiou exponential approach. They also employed an incompressible LBM to prevent the numerical instability commonly encountered in simulations with non-Newtonian fluids. This study focused primarily on analyzing the regularization parameter in a Bingham fluid simulation and proposes a method for determining it. To validate their model, the authors investigated pressure-driven planar channel and sudden expansion flows. The velocity profiles obtained from the pressure-driven planar channel flow agreed well with the analytical solutions. Furthermore, the calculated reattachment lengths for a 2:1 planar sudden expansion flow aligned well with existing data. Finally, the study explored the Bingham flow in a cavity, by examining streamlines and discussing yielded/unyielded regions.

Kefayati and Huilgol [27] conducted a study on the steady mixed convection in a square enclosure filled with a Bingham fluid. They employed both the Bingham and regularized Papanatasiou models, utilizing an innovative modification of the LBM. This investigation analyzed the effects of the yield stress on the heat and momentum transport by varying the Reynolds, Prandtl, and Bingham numbers. The results indicated that higher Reynolds numbers enhanced heat transfer and influenced the unyielded section, whereas higher Bingham numbers decreased heat transfer and increased the size of the unyielded section. Variations in the Prandtl number primarily affected the heat transfer. A comparison between the Bingham and Papanatasiou models revealed noticeable differences in the yield and unyielded sections.

Finally, the study conducted by Kefayati and Huilgol [28] investigated the steady flow in a square cross-section pipe filled with a Bingham fluid. This problem was solved using the Bingham model without regularization. Additionally, an innovative modification of the LBM was employed. The investigation focused on examining the effects of yield stress on momentum transport, while considering the Oldroyd number (Od = 0.1–0.53) and hydraulic diameter (DH = 1 and 2) as the relevant parameters.

Over the past few decades, numerous research studies have been conducted to enhance our understanding of the grouting mechanism. Despite these efforts, establishing a precise correlation between sealing efficiency and various influencing factors remains challenging.

Our contribution is significant as we employed the LBM, incorporating a modification to the relaxation parameter. This approach differs from that proposed by Sun et al. [14], offering an alternative to traditional Computational Fluid Dynamics (CFD) methods for simulating Bingham-type fluids. The rheological properties used in our LBM simulations were derived from experimental data of a water-cement mixture, and their accuracy was verified through analytical solutions.

We analyzed the behavior of these mixtures in a rectangular cavity with varying configurations of sudden expansion and contraction, which are commonly found in fractured rocks. This analysis yielded velocity distributions, streamlines, velocity profiles, and pressure distributions. These findings are crucial for understanding the mechanisms involved in grouting injection.

## 2 Constitutive equations of fluid motion

The continuity and momentum equations are two fundamental equations in fluid mechanics. The continuity equation is derived from the principle of mass conservation and is expressed as follows:

(1) $$\frac{\partial \rho }{\partial t}+\frac{\partial \left(\rho u_{i}\right)}{\partial x_{i}}=0$$

in the Eq. (1), ρ represents the density, and u_i_ represents the fluid velocity.

Eq. (2) is a mathematical representation of Newton's second law and is derived from the principle of momentum conservation. This implies that the momentum of a system remains constant when the total force acting on it is zero.

(2) $$\rho \left(\frac{\partial u_{j}}{\partial t}+u_{i}\frac{\partial u_{j}}{\partial x_{i}}\right)=-\frac{\partial \tau_{ij}}{\partial x_{i}}-\frac{\partial P}{\partial x_{j}}+\rho g_{j}$$

in the case of a Newtonian fluid, the shear stress ratio ($\tau_{ij}$) and strain rate tensor (${\dot{e}}_{ij}$) are related through the following expression.

(3) $$\tau_{ij}=2\mu {\dot{e}}_{ij};\;{\dot{e}}_{ij}=\frac{1}{2}\left(\frac{\partial u_{i}}{\partial x_{j}}+\frac{\partial u_{j}}{\partial x_{i}}\right)$$

By incorporating Eq. (3) into Eq. (2), we obtain Eq. (4), corresponding to the Navier–Stokes equation:

(4) $$\rho \left(\frac{\partial u_{j}}{\partial t}+u_{i}\frac{\partial u_{j}}{\partial x_{i}}\right)=\mu \frac{\partial^{2}u_{j}}{\partial x_{i}^{2}}-\frac{\partial P}{\partial x_{j}}+\rho g_{j}$$

in the case of a Bingham non-Newtonian fluid, the relationship between $\tau_{ij}$ and ${\dot{e}}_{ij}$ is given by the following equation.

(5) $$\tau_{ij}=2\mu_{\text{eff}}{\dot{e}}_{ij};\;\mu_{\text{eff}}=\frac{1}{2}\tau_{0}{\dot{e}}_{ij}^{-1}+\mu_{B}\text{,}$$

in Eq. (5), μ_eff_ represents the effective viscosity, and τ_0_ denotes the yield stress. In the absence of yield stress, the fluid behaves as a Newtonian fluid, meaning τ_0_ = 0 [24].

## 3 Lattice Boltzmann method (LBM)

The LBM was derived using the lattice gas automata (LGA) method. This methodology follows a similar updating procedure as the LGA method; however, instead of individually tracking "particles" as they move along lattice links, it focuses on the analysis of the distribution function, $f_{i}=\left(\vec{x},t\right)$. Hence, we assessed the evolution of $f_{i}=\left(\vec{x},t\right)$ by discretizing the Boltzmann equation on a lattice. The methodology can be summarized in two stages. The first stage involves the advancement of particles to the subsequent lattice site along the directions of motion for each time step Δt. The second stage involves simulating the collisions or interactions between the particles. Both stages can be described using the Boltzmann equation, as explained by Thürey [29] and Chen and Doolen [30].

(6) $$f_{i}\left(\vec{x}+{\vec{e}}_{i},t+1\right)-f_{i}\left(\vec{x},t\right)=-\Omega_{i}$$

The collision term Ω_i_ on the right-hand side of Eq. (6) generally takes a complex integral form. In various applications of the kinetic theory in fluid dynamics, efforts have been made to approximate the integral collision terms in simpler forms. One such example is the Bhatnagar–Gross–Krook (BGK) approach, where the collision term is replaced by the term, $\Omega_{BGK}=\left(f_{i}^{eq}-f_{i}\right)/\tau$. This operational model captures the effect of collisions as a relaxation process, driving the distribution towards the Maxwell equilibrium distribution, $f_{i}^{eq}=\left(\vec{x},t\right)$. The relaxation time (τ) has units of time, and it governs the frequency at which the distribution function relaxes to achieve a balance. In other words, this time parameter determines the rate at which fluctuations in the system reach a steady state. The BGK approach is commonly referred to as a single relaxation time approximation. Therefore, the discretized LBM equation incorporates the BGK approximation as follows:

(7) $$f_{i}\left(\vec{x}+{\vec{e}}_{i},t+1\right)-f_{i}\left(\vec{x},t\right)=-\frac{1}{\tau }\left[f_{i}\left(\vec{x},t\right)-f_{i}^{eq}\left(\vec{x},t\right)\right]$$

The macroscopic variables can be directly calculated from the values of the distribution function as shown in Eq. (8).

$$\rho \left(\vec{x},t\right)=\sum_{i=1}^{n}f_{i}\left(\vec{x},t\right)$$

(8) $$\vec{u}\left(\vec{x},t\right)=-\frac{1}{\rho \left(\vec{x},t\right)}\sum_{i=1}^{n}f_{i}\left(\vec{x},t\right){\vec{e}}_{i}$$

Discrete macroscopic pressure can be determined by an equation that relates the discrete density to the pressure through a simple proportional relationship, while considering the speed of sound. This relationship can be expressed as $P=c_{s}^{2}\rho \left(\vec{x},t\right)=\rho \left(\vec{x},t\right)/3$. In the case of a Newtonian fluid, the relationship between kinematic viscosity and relaxation time is given by $\upsilon =1/3\left(\tau -\frac{1}{2}\right)$, as described by Usman et al. [31].

One of the contributions of the method presented in this study is the modification of the lattice BGK approximation, as originally proposed by Wang and Ho [23] and Tang et al. [26], for simulating Bingham plastic fluids using the LBM with a D2Q9 model. This modification involves proposing a relaxation parameter τ as a function of apparent viscosity.

(9) $$\tau =3\left[\mu_{B}+\tau_{0}\left({\dot{e}}_{ij}\right)\right]+\frac{1}{2}\text{,}$$

In Eq. (9), $\mu_{B}$ and $\tau_{0}$ represent the Bingham viscosity and yield stress, respectively. The parameter τ was incorporated into Eq. (7).

## 4 Simulation of Bingham-type fluid for the LBM using the experimental date of cement grout

In this study, two types of boundary conditions were employed to calculate the distribution function (fi). The conditions used included periodic conditions for the inflow and outflow and no-slip bounce-back conditions for static walls. The periodic conditions involve maintaining the constant values of the distribution functions at one end and equalizing them to those at the opposite end. Their implementation in the LBM algorithm occurred during the propagation step, ensuring that the distribution functions at the outlet were identical to those at the inlet. That is, the values of the distribution functions at the outlet were equal to those at the inlet. In addition, bounce-back conditions were implemented when a static wall was encountered. These conditions imply that the distribution functions, upon colliding with a solid wall, retreat in the same direction but in the opposite sense, without losing energy. To ensure this, the collision was assumed to be elastic. Simulations were performed using a lattice size of 64 × 64. After 200,000 iterations, a steady-state was achieved.

To validate the proposed LBM, the results were compared with analytical solutions for Poiseuille flow between two plates separated by a distance of 2H.

(a) $$\frac{v_{x}}{v}=Bn\left(\frac{y}{H}-1\right)-\left(\frac{\Delta P}{L}\right)\frac{H^{2}}{2v\mu_{B}}\left[1-{\left(\frac{y}{H}\right)}^{2}\right]$$

(b) (10) $$\frac{v_{xp}}{v}=\left(-\frac{\Delta P}{L}\right)\frac{H^{2}}{2v\mu_{B}}\left(1-\frac{y_{p}}{H}\right)$$

Eq. (10a) represents the velocity profile for a Bingham fluid, where ν denotes the characteristic velocity, and Bn stands for the Bingham number, given by $Bn=\tau_{0}H/\mu_{B}v$, and (ΔP/L) represents the pressure drop. Eq. (10b) defines the corresponding velocity ν_xp_, specifically within the plug region.

In this study, we used the results obtained by Liu et al. [14] from the rheological characterization of cement grout. They found a grout flow pattern consistent with Bingham-type fluids, with an initial yield stress (τ_0_) of 2.615 Pa and a plastic viscosity (μ_B_) of 0.0175 Pa s at 20 °C. The Bingham number was calculated to be 0.498.

In Table 1, the conditions utilized for the simulations are presented for the Bingham numbers of 0.1, 0.2, 0.3, 0.4, and 0.498, expressed in units of lattice (u.l.).

**Table 1:** Conditions employed for the simulation of the LBM for Bingham-type fluids.

Bingham number	Yield stress (u. l.)	Force (u. l.)	Bingham viscosity (u. l.)
0.1	2.00E-5	2.66E-2	0.400
0.2	1.10E-5	5.83E-3	0.080
0.3	1.40E-5	5.19E-3	0.070
0.4	6.50E-6	1.88E-3	0.025
0.498	9.20E-6	2.34E-3	0.011125

**Fig. 1:**
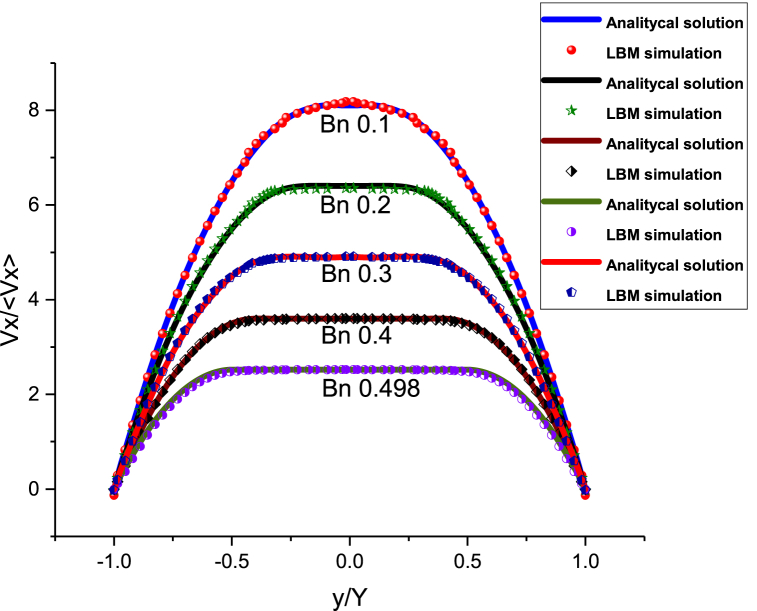
Comparison of the LBM simulations with the analytical solutions for the five Bingham numbers. The error percentage is less than 1.7%.

In Fig. 1, the simulation results of the LBM using four Bingham numbers are compared with the analytical solutions. The error percentage was found to be less than 1.7%.

**Fig. 2:**
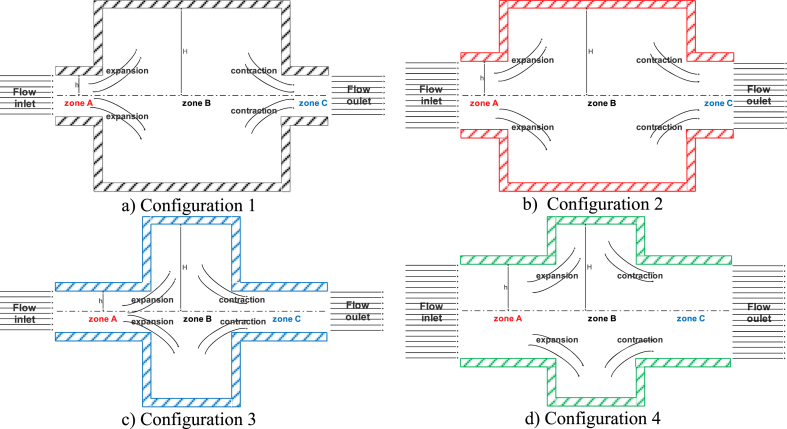
Ducts with a rectangular geometry featuring both expansion and contraction.

Four configurations, consisting of rectangular ducts to represent certain types of rock fractures, were proposed, as shown in Fig. 2. Simulations were conducted in the ducts using cement grout (referred to as fluid I hereafter) and a Newtonian fluid with similar characteristics to cement grout but without yield stress (referred to as fluid II). The objective of these simulations was to analyze the behavior of both fluids in response to expansion and contraction.

The ducts are divided into three zones. Zone A represents the fluid inlet to the duct, zone B represents the expansion, and zone C represents the contraction. The Reynolds number was calculated using the formula, #Re=ρhv‾μB. Here, h is the characteristic length, which is half the channel width before expansion (Fig. 2); ρ is the density; $\bar{v}$ denotes the average velocity; and $\mu_{B}$ represents the Bingham viscosity.

In our experiments, we confirmed the formation of vortices for fluid I with Reynolds numbers greater than 10, as reported by Missirlis et al. [32], Halmos and Boger [33], and Manica and De Bortoli [20] for non-Newtonian fluids. The results are presented in Table 2 in lattice units.

**Table 2:** Summary of the simulations for fluids I and II across four configurations in lattice units (u.l.).

			Pressure force	Average velocity	# Re	Yield stress
Configuration 1	Fluid I	viscosity = 1.1125E-2	2.3406E-3	2.2621E-3	1.0167	9.2E-6
			2.3406E-2	3.2597E-2	14.6504	
	Fluid II		2.3406E-3	4.4690E-3	2.0085	0.0
			2.3406E-2	3.3807E-2	15.1942	
Configuration 2	Fluid I		2.3406E-3	1.8830E-2	16.9259	9.2E-6
			5.0E-3	4.2633E-2	38.3216	
	Fluid II		2.3406E-3	2.3177E-2	20.8335	0.0
			5.0E-3	4.6952E-2	42.2041	
Configuration 3	Fluid I		2.3406E-3	2.2739E-3	1.0219	9.2E-6
			2.3406E-2	4.0906E-2	18.3846	
	Fluid II		2.3406E-3	4.6766E-3	2.1018	0.0
			2.3406E-2	4.2934E-2	19.2963	
Configuration 4	Fluid I		2.3406E-3	4.0442E-2	47.2585	9.2E-6
			8.0E-3	1.5599E-1	182.2842	
	Fluid II		2.3406E-3	4.9128E-2	57.4080	0.0
			8.0E-3	1.6502E-1	192.8350	

**Fig. 3:**
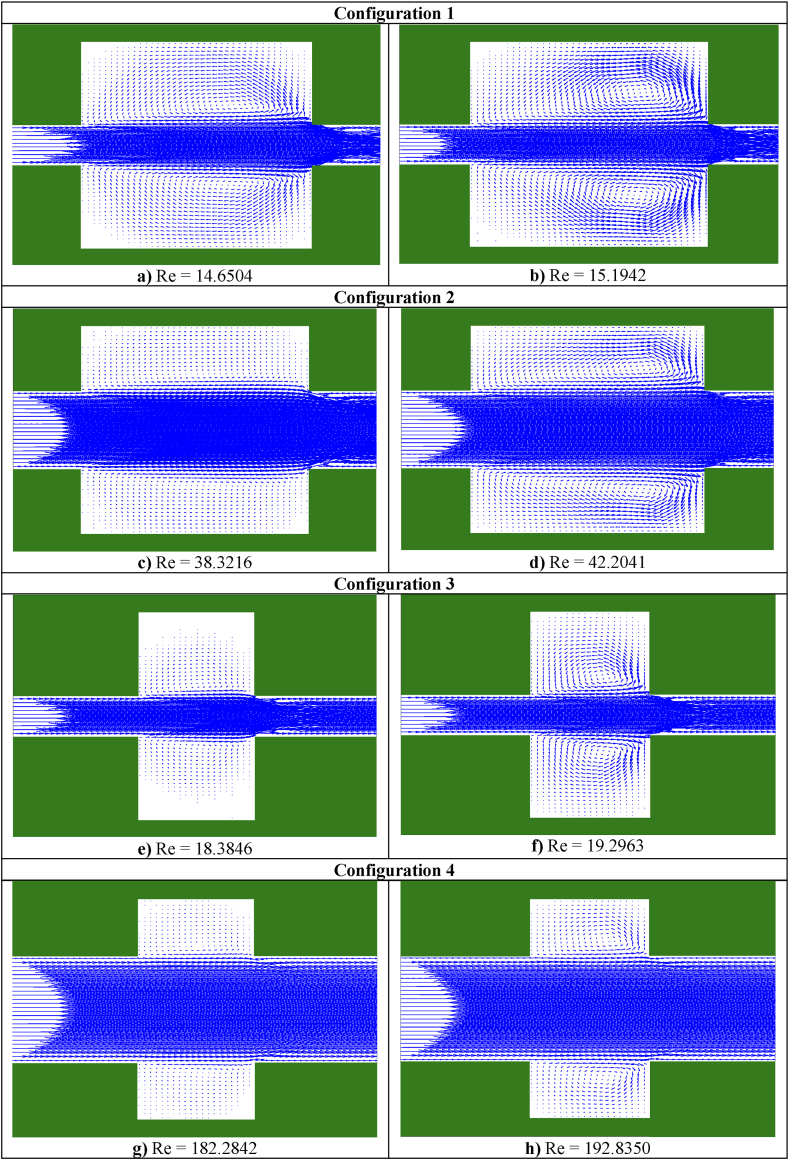
Streamlines of cement grout and uncemented water for the four configurations. Configuration 1 consists of: **a)** Fluid I with viscosity = 1.1125E-2, force pressure = 2.3406 E−2 and yield stress 9.2E-6 and **b)** Fluid II with viscosity = 1.1125E-2 and force pressure = 2.3406 E−2. Configuration 2 includes: **c)** Fluid I with viscosity = 1.1125E-2, force pressure = 5.0E-3, and yield stress 9.2E-6 and **d)** Fluid II with viscosity = 1.1125E-2 and force pressure = 5.0E-3. Configuration 3 encompasses: **e)** Fluid I with viscosity = 1.1125E-2, force pressure = 2.3406 E−2, and yield stress 9.2E-6 and **f)** Fluid II with viscosity = 1.1125E-2 and force pressure = 2.3406 E−2. Configuration 4 is comprised of: **g)** Fluid I with viscosity = 1.1125E-2, force pressure = 8.0E-3, and yield stress 9.2E-6 and **h)** Fluid II with viscosity = 1.1125E-2 and force pressure = 8.0E-3.

A summary of the results from Table 2, showing the behaviors of the two types of fluids, is presented in Fig. 3. In both cases, vortex formation was observed in the expansion and contraction zones, with the effect being more pronounced in fluid II. In addition, the phenomenon of vena contracta occurred in both cases.

In Fig. 3, the flows of fluids I (Fig. 3a) and II (Fig. 3b) in configuration 1 are depicted under similar conditions. A difference in the behavior of the fluids was clearly observed through the streamlines of both types of fluids, and the streamlines were more intense for fluid II. In the case of fluid I, the formation of vortices was observed slightly to the right of the central zone, slightly below and above the centerline of the duct. In contrast, for fluid II, vortex formation was more intense, and vortices were observed to the right, upward, and downward from the central zone. Fig. 3c and d depict the passage of fluids I and II, respectively, through configuration 2 under similar conditions. In this configuration, the duct entry has been duplicated. Hence, the behavior of the fluids can be clearly observed. Here, the fluid behavior is evident from the streamlines. These streamlines are more intense in fluid II. In Fig. 3c, vortex formation occurs in the central zone, slightly below and above the centerline of the duct. However, in Fig. 3d, the vortex formation is more intense and is observed to the right, upward, and downward of the central zone. Fig. 3e and f shows the passage of fluids I and II, respectively, through configuration 3 under similar conditions, where the duct entry and zone B are reduced. Similarly, the behavior of the fluids can be clearly observed through the streamlines. These streamlines are more intense in fluid II. In Fig. 3e, vortex formation occurring in the central zone, slightly below and above the centerline of the duct, is more notable. In Fig. 3f, the vortex formation is more intense, and they move slightly to the right, downward, and upward from the central zone. Furthermore, Fig. 3g and h correspond to the passage of fluids I and II, respectively, through configuration 4 under similar conditions. Here, the duct entry is increased and zone B is reduced. The behavior of the fluids can be clearly observed through the streamlines. These streamlines are more intense in fluid II. In Fig. 3g, the vortices formed in the central zone, slightly below and above the centerline of the duct. In Fig. 3h, the formation of vortices is more intense, and they move slightly to the right, downward, and upward from the central zone.

Variations in the distribution of flow velocities and pressures can be observed when there is a sudden expansion or contraction in the conduit geometry. In the first case, when a fluid flows from a smaller section to a larger section, the velocities or flow lines decrease abruptly, leading to turbulence and energy loss [34]. In the second case, when the flow transitions from a larger to a smaller section, the fluid currents contract and adapt to a curved path over a certain distance. Consequently, the effective minimum cross-sectional area of the flow becomes smaller. This minimum flow area is known as the "vena contracta," and this requires the flow to decelerate and expand to fill the conduit.

**Fig. 4:**
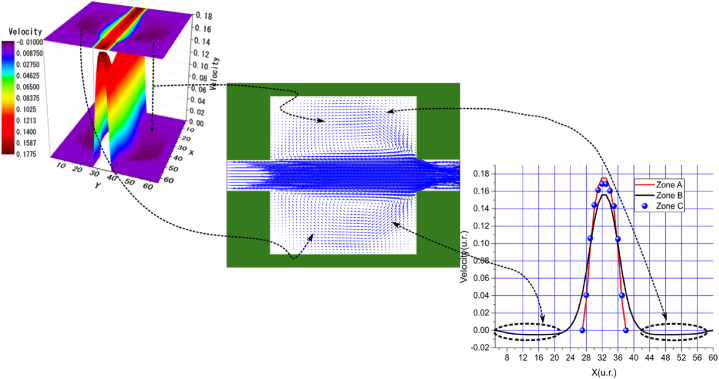
Velocity distribution, streamlines, and velocity profiles for cement grout. Force = 2.3406E-2, yield stress = 9.2E-6, viscosity = 1.1125E-2, and average speed = 3.2597E-2 (in unit of lattice (u.l.)).

**Fig. 5:**
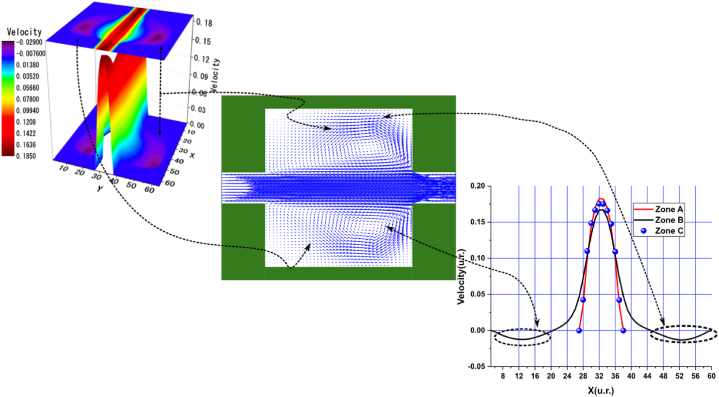
Velocity distribution, streamlines, and velocity profiles for uncemented water. Force = 2.3406E-2, viscosity = 1.1125E-2, and average speed = 3.3807E-2 (in unit of lattice (u.l.)).

**Fig. 6:**
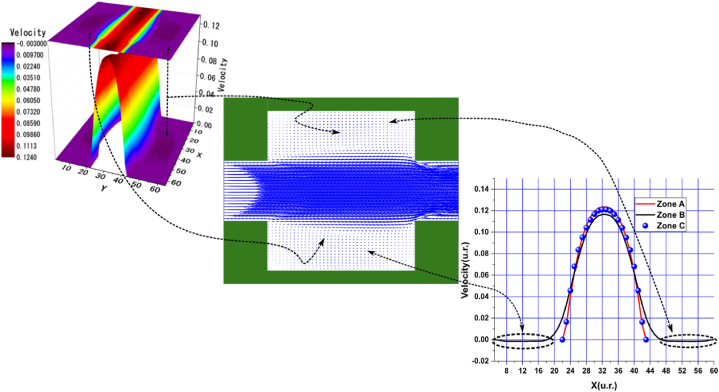
Velocity distribution, streamlines, and velocity profiles for cement grout. Force = 5.0E-3, yield stress = 9.2E-6, viscosity = 1.1125E-2, and average speed = 4.2633E-2 (in unit of lattice (u.l.)).

**Fig. 7:**
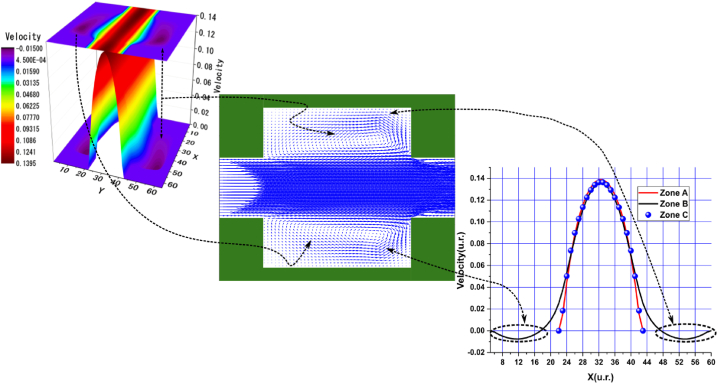
Velocity distribution, streamlines, and velocity profiles for uncemented water. Force = 5.0E-3, viscosity = 1.1125E-2, and average speed = 4.6952E-2 (in unit of lattice (u.l.)).

**Fig. 8:**
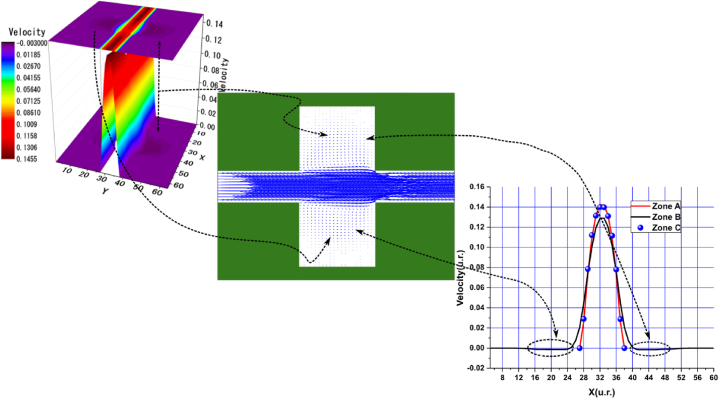
Velocity distribution, streamlines, and velocity profiles for cement grout. Force = 2.3406E-2, yield stress = 9.2E-6, viscosity = 1.1125E-2, and average speed = 4.0906E-2 (in unit of lattice (u.l.)).

**Fig. 9:**
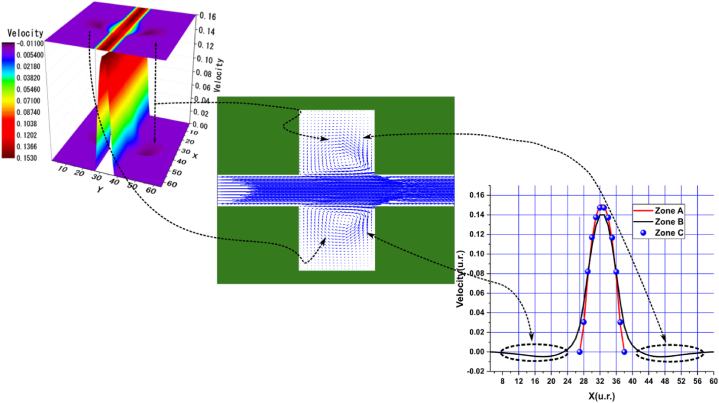
Velocity distribution, streamlines, and velocity profiles for uncemented water. Force = 2.3406E-2, viscosity = 1.1125E-2, and average speed = 4.2934E-2 (in unit of lattice (u.l.)).

**Fig. 10:**
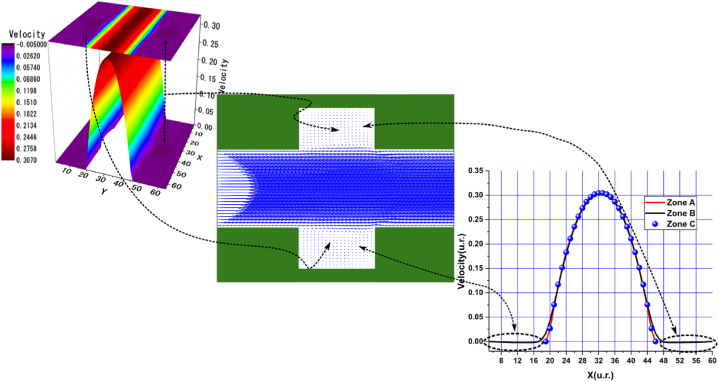
Velocity distribution, streamlines, and velocity profiles for cement grout. Force = 8.0E-3, yield stress = 9.2E-6, viscosity = 1.1125E-2, and average speed = 1.5599E-1 (in unit of lattice (u.l.)).

**Fig. 11:**
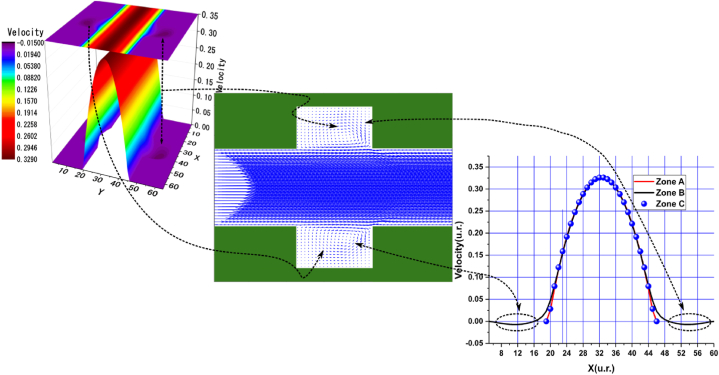
Velocity distribution, streamlines, and velocity profiles for uncemented water. Force = 8.0E-3, viscosity = 1.1125E-2, and average speed = 1.6502E-1 (in unit of lattice (u.l.)).

In the arrangements used, we observed various sudden expansions and contractions, with the most pronounced expansions occurring in Arrangements 1 and 3, followed by Arrangements 2 and 4. According to these arrangements, the vortex formation can be clearly observed in the velocity profile graphs near the nodes of the upper and lower walls, corresponding to the black curve in zone B. In these graphs, the velocities are negative due to the presence of vortices. This phenomenon is particularly evident in the central streamline graphs shown in Fig. 4, Fig. 5, Fig. 6, Fig. 7, Fig. 8, Fig. 9, Fig. 10, Fig. 11.

**Fig. 12:**
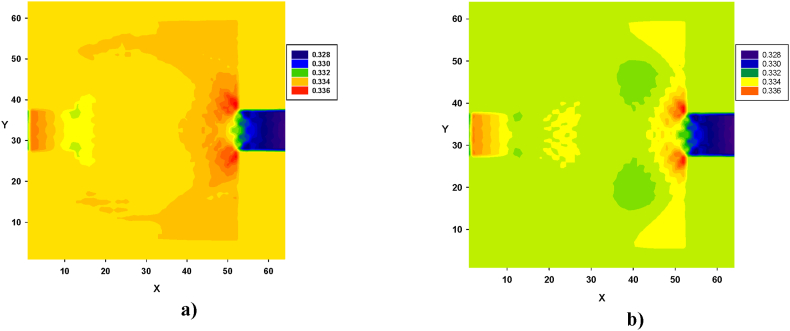
Pressure distribution in configuration 1, for yield stress = 9.2E-6, viscosity = 1.1125E-2, and force = 2.3406E-2. **a)** Fluid I, Re = 14.6504 and **b)** fluid II, Re = 15.1942 (in unit of lattice (u.l.)).

**Fig. 13:**
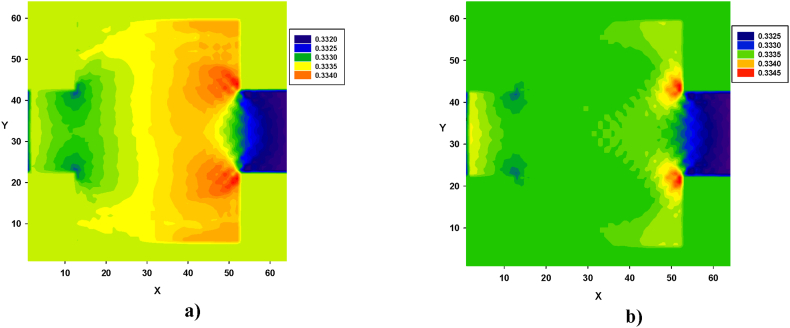
Pressure distribution in configuration 2, for yield stress = 9.2E-6, viscosity = 1.1125E-2, and force = 5.0E-3. **a)** Fluid I, Re = 38.3216 and **b)** fluid II, Re = 42. 2041 (in unit of lattice (u.l.)).

**Fig. 14:**
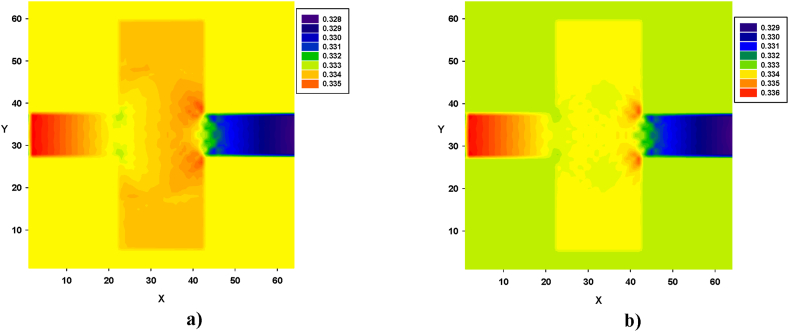
Pressure distribution in configuration 3, for yield stress = 9.2E-6, viscosity = 1.1125E-2, and force = 2.3406E-2. **a)** Fluid I, Re = 18.3846 and **b)** fluid II, Re = 19.2963 (in unit of lattice (u.l.)).

Turbulence, caused by contraction and expansion, leads to energy loss. This effect is illustrated in Fig. 12, Fig. 13, Fig. 14. Pressure contours were obtained for both types of fluids, in all four configurations. Similarly, in Fig. 12, Fig. 13, Fig. 14, for both types of fluids, the pressure decreased from left to right. In all four configurations, the regions of high-pressure stagnation, observed near the corners at the end of zone B, were more pronounced in fluid I than in fluid II. Furthermore, owing to the pressure drops caused by the formation of vortices, which were more pronounced in fluid II for all four configurations, the relationship between the velocity and pressure gradient was not linear, exhibiting a Forchheimer flow [35].

Significant differences in the pressure distribution are observed for fluid I in Fig. 12, along with the expansion of zone B. Similar variations can be seen in the center of that area, although they deviate from the central line of the conduit. In addition, pressure variations were observed at the corners of the section, particularly at the end of zone B, as shown in Fig. 12a. For fluid II, significantly lower pressure values were observed throughout the final part of zone B, as shown in Fig. 12b.

As shown in Fig. 13, variations in the pressure distribution for fluid I can be observed along zone B, with more prominent differences observed at the end of zone B, especially in the corners, as depicted in Fig. 13a. Similarly, for fluid II, lower pressure values were prominently distributed across the width of the final part of the zone, as shown in Fig. 13b.

In Fig. 14, variations in the pressure distribution for fluid I can be observed in zone B, particularly across the width at both the beginning and end of the zone, as shown in Fig. 14a. In contrast, for fluid II, lower pressure values were prominently distributed across both the width and length of zone B, as depicted in Fig. 14b.

**Fig. 15:**
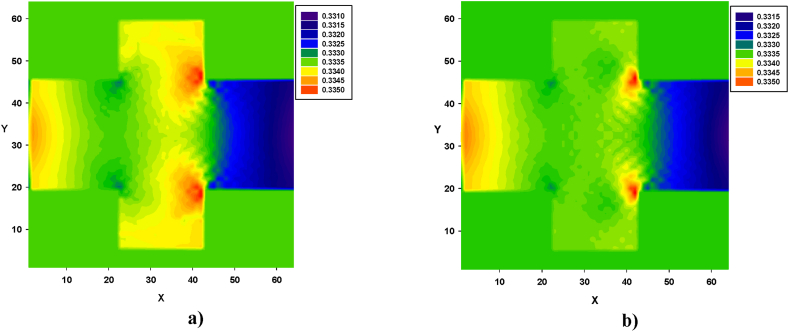
Pressure distribution in configuration 4, for yield stress = 9.2E-6, viscosity = 1.1125E-2, and force = 8.0E-3. **a)** Fluid I, Re = 182.2842 and **b)** fluid II, Re = 192.8350, in unit of lattice (u.l.).

Finally, in Fig. 15, variations in the pressure distribution for fluid I can be observed along the length and width of zone B, with more significant differences observed at the end of the zone, especially in the corners, as depicted in Fig. 15a. In the case of fluid II, lower pressure values were prominently distributed across the width of the final part of the zone, as shown in Fig. 15b.

## 5 Conclusions

The simulations performed in this study clearly demonstrated the formation of vortices in the expansion and contraction zones for both types of fluids, with a more intense effect observed in fluid II. Additionally, the presence of the "vena contracta" phenomenon was confirmed in both configurations.

Significant differences in the behaviors of the two types of fluids were observed in all four configurations. Fluid II exhibited a more intense vortex formation than fluid I.

In configuration 1, the vortices were more intense for fluid II, extending to the right, upward, and downward from the central zone of the conduit.

In configuration 2, with duplicated fluid entry, the formation of more intense vortices in fluid II was reiterated, both in the central zone and below and above the conduit centerline.

In configuration 3, in which the conduit entry and zone B were reduced, the streamline patterns showed more intense vortices for fluid II. For fluid I, the formation of vortices was more notable in the central zone and slightly below and above the centerline of the conduit, whereas for fluid II, the vortices were more intense, and they moved slightly to the right, downward, and upward from the central zone.

In configuration 4, where the conduit entry was increased and Zone B was reduced, the streamline patterns showed more intense vortices in fluid II. For fluid I, the vortices formed in the central zone and slightly below and above the centerline of the conduit, whereas for fluid II, the vortices, which were more intense, moved slightly to the right, downward, and upward from the central zone.

In summary, in all configurations, fluid II exhibited a more intense vortex formation than fluid I, indicating a distinct behavior between both types of fluid under the same viscosity and pressure force conditions but with different configuration conditions.

Similarly, pressure contours were obtained for both types of fluids, in all four configurations. The results revealed that in both cases, the pressure decreased from left to right. In all configurations, the regions of high-pressure stagnation were observed near the corners at the end of zone B, and these were more pronounced in fluid I than in fluid II. Furthermore, owing to the pressure drops caused by the formation of vortices, which were more pronounced in fluid II for all four configurations, the relationship between the velocity and pressure gradient was not linear, with a Forchheimer flow [27].

These findings provide valuable insights into the behavior of fluids during the contraction and expansion processes. The presence of high-pressure stagnation regions and nonlinear relationship between the velocity and pressure gradients have significant implications for various engineering applications. Understanding the pressure distribution in these scenarios is fundamental for optimizing the processes and reducing energy losses.

In summary, the reinforcement behavior of fractured rocks with cement grout exhibiting rheological characteristics akin to a Bingham fluid is investigated through simulation using the Lattice Boltzmann Method, incorporating a modification to the relaxation parameter. This approach differs from that proposed by Sun et al. [14]. The rheological characteristics employed in our LBM simulations were obtained from experimental findings involving a water-cement blend, and their precision was validated using analytical methods, as well as for a fluid without cement (Newtonian fluid). The effects of the rheological parameters of the Bingham plastic fluid were analyzed in a rectangular cavity with different configurations of sudden expansion and contraction, commonly found in fractured rocks.

From our computational findings, several conclusions can be inferred:

Our computational experiments revealed that the intensity of vortices generated in expansive and contractive regions is directly related to the yield stress value. In particular, we observed that vortices are more intense in a fluid with zero yield stress, like Newtonian fluid, compared to Bingham fluid, resulting in pressure drops. Consequently, the disparity in the more pronounced vortex formation between Newtonian fluids and Bingham plastics is attributed to the yield stress term. It was observed that Newtonian fluids, lacking this term, exhibit a higher intensity of vortices compared to Bingham fluids.

The obtained results offer valuable contributions for their application in geotechnical engineering. In addition, this analysis provided essential information on velocity distributions, streamlines, velocity profiles, and pressure distributions. These results are fundamental for comprehending the processes involved in grouting injection, with the potential to optimize processes and reduce time and costs. It has been demonstrated that the variables of utmost importance are related to the rheological parameters of the fluids. These insights acquired in the field of fluid dynamics and engineering applications are relevant for future research and process optimization.

The ultimate goal is to propose a predictive model based on mortar reinforcement for fractured rocks, taking into account rheological properties and water-cement ratio, thus reducing the need for costly experiments.

## Data availability

The data used in this study can be availed from the corresponding author upon request.

## CRediT authorship contribution statement

**José Luis Velázquez Ortega:** Writing – review & editing, Supervision, Methodology, Investigation, Conceptualization. **Alberto Ignacio Guerrero Vergara:** Validation, Methodology, Investigation.

## Declaration of competing interest

The authors declare that they have no known competing financial interests or personal relationships that could have appeared to influence the work reported in this paper.

## Nomenclature

*ρ*: Density
*t*: Time
*u*_*i*_*, u*_*j*_: Velocity
*x*_*i*_*, x*_*j*_: Position
*τ*_*ij*_: Shear stress
*μ*: Viscosity
*ė*_*ij*_: Strain rate tensor
*P*: Pressure
*g*_*i*_: Gravity
*μ*_*eff*_: Effective viscosity
*τ*_*0*_: Yield stress
*μ*_*B*_: Bingham viscosity
*v*_*x*_: Velocity profile
*v*: Characteristic velocity
*Bn*: Bingham number
*ΔP/L*: Pressure drop
*v*_*xp*_: Velocity in the plug region
*y*_*p*_: Position in the plug region
*y*: Position on the plates
*H*: Half the distance between the plates
*#Re*: Reynolds number
$\bar{v}$: Average velocity
*h*: Characteristic length
$f_{i}$: Lattice Boltzmann distribution function
$f_{i}^{eq}$: LB equilibrium distribution function
$\vec{x}$: Position
${\vec{e}}_{i}$: Discrete unit velocities
Ω_i_: Collision operator
$\Omega_{BGK}$: Collision operator model, BGK approach
τ: LB relaxation time
$\vec{u}\left(\vec{x},t\right)$: Macroscopic velocity
$\rho \left(\vec{x},t\right)$: Macroscopic density
P: Pressure
υ: Kinematic viscosity
